# Comparison of hybrid coronary revascularization versus coronary artery bypass grafting in patients with multivessel coronary artery disease: a meta-analysis

**DOI:** 10.1186/s13019-022-01903-w

**Published:** 2022-06-07

**Authors:** Li Yu, Keying Zhu, Nannan Du, Yuexiu Si, Jiali Liang, Ruijing Shen, Bangsheng Chen

**Affiliations:** 1 Department of Cardiology, Ningbo Yinzhou No. 2 Hospital, Ningbo, Zhejiang China; 2 Clinical Medicine Science, The Second Clinical Medical College, Zhejiang Chinese Medical University, Hangzhou, Zhejiang China; 3 Biochemistry Laboratory, School of Basic Medical Sciences, Zhejiang Chinese Medical University, Hangzhou, Zhejiang China; 4 Clinical Medicine Science, The First Clinical Medical College, Zhejiang Chinese Medical University, Hangzhou, Zhejiang China; 5 Emergency Medical Center, Ningbo Yinzhou No. 2 Hospital, 998 North Qianhe Road, Yinzhou District, Ningbo, 315100 Zhejiang China

**Keywords:** HCR, CABG, MACCE, MVD, Meta-analysis

## Abstract

**Background:**

Percutaneous coronary intervention (PCI) and coronary artery bypass grafting (CABG) are widely used in the treatment of coronary heart disease, but the best revascularization method for multivessel coronary artery disease (MVD) patients is still controversial. Hybrid coronary revascularization (HCR), together with CABG and PCI, have been proved to be feasible methods, but the long-term effect of HCR is not as clear as CABG.

**Method:**

By October 2020, we retrieved articles from PubMed, Web of science, EMBASE and Cochrane library databases. The main results are based on major adverse cardiovascular and cerebral events (MACCE).

**Result:**

A total of 18 articles (3 randomized controlled trials (RCTs) and 15 observational studies) were included in this meta-analysis. The outcomes of MACCE in the HCR group at perioperative, short-term (30 days to 1 year), medium-term (1 year to 5 years) and long-term (5 years and above) follow-up period were similar to those in the CABG group. The mortality rates of patients in perioperative, short-term and medium-term follow-up were similar to those in the CABG group, but lower than that in the CABG group at long-term follow-up (OR = 0.35, 95% CI 0.18–0.69, p = 0.002). The revascularization rate was higher in the HCR group during the perioperative period (OR = 3.50, 95% CI 2.07–5.94, p < 0.001), short-term (OR = 3.28, 95% CI 1.62–6.64, p < 0.001) and mid-term follow-up (OR = 2.84, 95% CI 1.64–4.92, p < 0.001).

**Conclusion:**

Our results reveal that HCR is a safe and therapeutically effective alternative in treatments for MVD patients. It has not only less short-term adverse effect, but also better long-term effect, especially in death.

**Supplementary Information:**

The online version contains supplementary material available at 10.1186/s13019-022-01903-w.

## Introduction

According to the World Health Organization report, in the 2016, Cardiovascular diseases (CVDs) are the number one cause of mortality globally, killing approximately 17.9 million each year [1]. Coronary artery disease (CAD), which can present as angina pectoris, myocardial infarction (MI), and ischemic heart failure [2], is one of the major types of CVDs [3]. Coronary artery bypass grafting (CABG) and percutaneous coronary intervention (PCI) are the two main treatments for patients with multivessel coronary artery disease (MVD) including left main coronary artery (LMCA) disease [2]. CABG results in a lower mortality & MI rate [4–6]. These benefits come from grafting of the left internal mammary artery(LIMA) to the left anterior descending (LAD) artery [7]. However, compared with PCI, CABG is a relatively invasive surgical procedure with a greater possibility of causing immediate complications such as bleeding, stroke, atrial fibrillation (AF), and eventually, prolonged hospitalization [2, 8–10]. PCI is a lesser invasive intervention, which allows the minimum procedural risk and a shorter recovery period. But it increases the possibility of repeat revascularization [2, 10, 11]. Therefore, the optimum revascularization strategy for MVD remains controversial [2, 12, 13].

Hybrid coronary revascularization (HCR), grafting LIMA to LAD along with PCI in the non-LAD vessels, is a less invasive alternative for MVD patients while maintaining durability. HCR may decrease repeat revascularization rate and enhance long term outcomes through benefiting from LIMA-LAD revascularization. As a minimum invasive intervention, HCR reduces risk of bleeding and infection, time of mechanical ventilation, and length of stay [7, 14].

In the recent years, HCR has received considerable attention as being the most suitable revascularization strategy for patients with MVD. In the current meta-analysis of HCR and CABG, no significant change in terms of in-hospital death, MI and stroke was observed between the two groups. Moreover, the need for red blood transfusion, length of hospitalization, length of intensive care unit (ICU) stay, and ventilation time were all better in HCR group [15–17]. HYBRID (Hybrid Revascularization for Multivessel Coronary Artery Disease) trial investigated a 5-year clinical follow-up of patient population that was randomly assigned to HCR group and CABG group. All-cause mortality available for entire cohort were similar in the 2 groups. No significant difference in the rates of MI, repeat revascularization, stroke and major adverse cardiovascular and cerebral events (MACCE) was observed in both the groups [18]. Regrettably, due to the lack of data in clinical trials and meta-analysis including numerous patients with long-term follow-up and various HCR operating conditions, HCR is not implemented broadly in clinical practice [19]. It was suggested that HCR is an alternative for CABG and PCI under specific circumstances listed in American College of Cardiology Foundation/American Heart Association guidelines and American College of Cardiology Foundation/American Heart Association guidelines [20, 21].

Several RCTs and observational studies have compared the long-term outcomes of HCR and CABG. Herein, a meta-analysis was conducted to compare the long-term difference between HCR and CABG in patients with MVD.

## Method

### Literature search strategy

Systematic literatures were retrieved using these databases following the Preferred Reporting Items for Systematic Reviews and Meta-Analyses (PRISMA) [22]: PubMed, Web of Science, Embase, and the Cochrane Library database. The search was updated until October, 2020. The research subject was people with CAD. The key words included were “coronary artery disease” OR “coronary disease” OR “cardiovascular disease” OR “heart disease” OR “myocardial infarction” OR “coronary syndrome” OR “multivessel coronary artery disease” OR “left main coronary artery disease” AND “hybrid coronary revascularization” OR “hybrid myocardial revascularization” OR “integrated myocardial revascularization” OR “hybrid revascularization” OR “integrted coronary revascularization” OR “hybrid revascularization” OR “hybrid coronary intervention” OR “hybrid percutaneous coronary intervention”OR “hybrid percutaneous intervention” OR “hybrid coronary artery revoscularization” AND “coronary artery bypass” OR “coronary artery bypass graft” OR “coronary artery bypass grafting” OR “CABG” or “coronary artery bypass surgery”. We supplemented the studies, trials, and review articles manually for potential additional studies. The primary outcomes being analyzed were perioperative, short-term (30 days to 1 year), mid-term (1 year to 5 years) and long-term (5 years and above) MACCE (MI, stroke, mortality, and repeat revascularization). Secondary outcomes focused in individual aspects, more specifically, each period MACCE and in-hospital outcomes.

### Inclusion and exclusion criteria

Studies that have met the following criteria were included: (1) original research that compared HCR and CABG for MVD patients; (2) RCTs or observational studies (cohort or case–control studies); (3) relevant data reported. Exclusion criteria were as followed: (1) ongoing studies or with unavailable data; (2) duplicate reports. The latest article will be included / selected if there were duplicate publications. (3) patient with previous experience of coronary revascularization. (4) The language was not English.

### Data extraction and quality assessment

Two independent authors evaluated the included RCTs and observational studies via the Cochrane Collaboration’s tool [23] and the Newcastle–Ottawa Quality Assessment Scale [24]. Disagreements were resolved by a third researcher. The following information was extracted: author, publication year, country, sample size, technique of HCR and CABG, extent of CAD, type of stent, staging strategy and study design.

### Statistical analysis

We used Review Manager 5.3 (The Cochrane Collaboration) to analyze the extracted data. The odds ratio (OR) and 95% confidence interval (CI) were calculated to establish dichotomous variables while the standardized mean difference (SMD) and the weighted mean difference (WMD) was were calculated for continuous variables. Heterogeneity among studies was quantified using I^2^ statistic. The fixed effects model was applied when p > 0.1 otherwise the random effects model would be used. p < 0.05 was considered statistically significant.

## Result

### Eligible studies

**Fig. 1:**
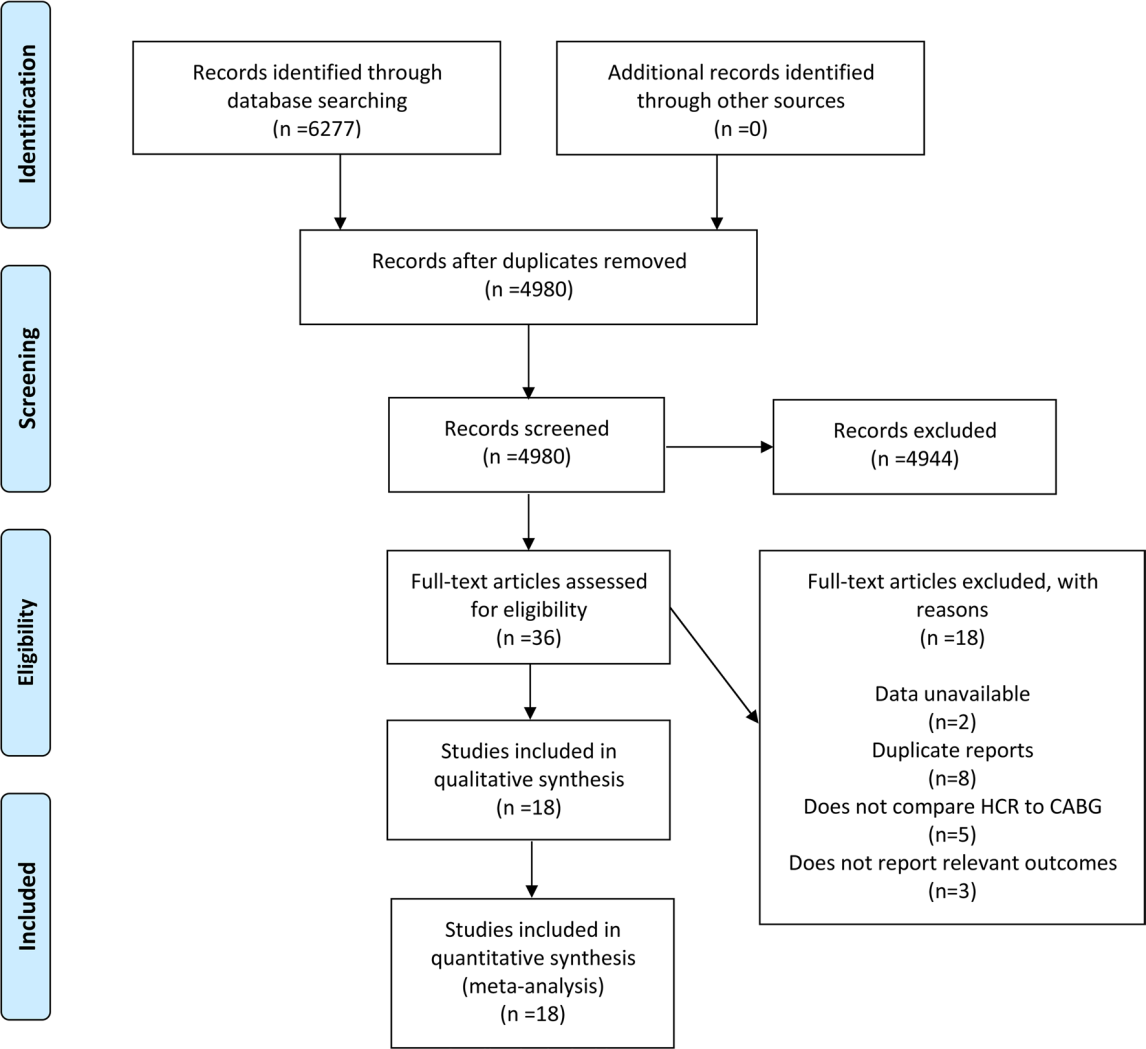
Flow chart of study selection

The search strategy originally generated 6,277 relevant clinical records from the four mentioned databases. There were eventually 18 studies (3 RCTs [25–27] and 15 observational studies [28–42]), which fulfilled our inclusion criteria after screening and eligibility assessment, thus were included in the analysis. The detailed searching and selecting process as well as the exclusion criteria can be found in Fig. 1.

### Study characteristics and quality

Basic characteristics of all the studies included are shown in Table 1. There were 2041 cases of HCR and 2993 cases of CABG. The majority of patients included had been suffering from MVD. Patients in the HCR group underwent staged or single-staged procedures and the most commonly used stent type was a drug eluting stent (DES). Of all the subjects in the HCR, only LAD bypass received the bypass grafting. Patients in CABG group received on-pump or off-pump procedures. Other detailed characteristics were presented in Additional file 1: Table S1. The RCTs and observational studies were assessed by the Cochrane Collaboration’s tool and Newcastle–Ottawa Quality Assessment Scale, respectively. The results of the risk bias assessment were shown in Additional file 2: Table S2.

**Table 1:** Characteristics of all the studies included in the meta-analysis

Author	Year	Country	Number of patients		HCR technique	CABG technique	Number of CAD	Extent of CAD	Stents	Staging strategy	Study design
			HCR	CABG							
Hage	2019	Canada	216	147	Robotic-assisted MIDCAB	OPCAB	Double-vessel CAD	LAD with non-LAD lesions	NA	NA	Cohort
Patel	2018	America	207	207	MIDCAB	CABG/OPCAB	Double-vessel CAD	LAD with circumflex or RCA	DES	Staged: CABG first	Cohort
Qiu	2019	China	52	128	NA	OPCAB	Double-vessel CAD	LAD with non-LAD lesions	DES	NA	Cohort
Wu	2017	China	73	383	NA	OPCAB	MVD	LAD with non-LAD lesions	DES	Staged: CABG first	Cohort
Di Bacco	2019	Italy	89	89	MIDCAB	NA	NA	NA	NA	Staged: PCI first	Cohort
Hannan	2020	American	302	302	MIDCAB	NA	MVD	LAD with non-LAD lesions	NA	Staged	Cohort
Shen	2013	China	141	141	MIDCAB	CABG/OPCAB	MVD	LAD with non-LAD lesions	DES	Single-stage	Cohort
Modrau	2020	Denmark	103	103	MIDCAB/OPCAB	CABG	MVD	LAD with non-LAD lesions	DES	NA	Cohort
Basman	2020	America	100	100	robotic-assisted MIDCAB	CABG/OPCAB	TVD	NA	DES	Staged: CABG first	Cohort
Zhao	2009	America	112	154	NA	CABG/OPCAB	NA	NA	NA	Single-stage	Cohort
Delhaye	2010	France	18	18	NA	CABG/OPCAB	MVD	LAD with non-LAD lesions	DES	Staged: CABG first	Cohort
Harskamp	2015	Netherlands	306	918	MIDCAB	NA	MVD	NA	DES	Staged	Cohort
Kon	2008	America	15	30	NA	OPCAB	MVD	LAD with non-LAD lesions	DES	Single-stage	Cohort
de Cannière	2001	America	20	20	MIDCAB + PTCA	CABG	Double-vessel CAD	LAD with non-LAD lesions	DES or BMS	Staged: PCI first	Cohort
Farid	2018	America	100	82	MIDCAB	MIDCAB	MVD	LAD with non-LAD lesions	DES or BMS	Staged: CABG first	Cohort
Gąsior	2014	Poland	98	102	MIDCAB/EACAB	CABG/OPCAB	MVD	LAD with non-LAD lesions	DES	NA	RCT
Ganyukov	2020	Russia	49	49	MIDCAB	CABG	MVD	LAD with non-LAD lesions	DES	Staged: CABG first	RCT
Esteves	2020	Brazil	40	20	NA	CABG	TVD	NA	NA	Staged: CABG first	RCT

CABG = coronary artery bypass grafting; HCR = Hybrid coronary revascularization; CAD = coronary artery disease; OPCAB = Off-pump coronary artery bypass grafting; MIDCAB = minimally invasive CABG; DES = drug-eluting stent; MVD = multivessel coronary artery disease; PTCA = percutaneous transluminal coronary angioplasty; BMS = bare metal stent; EACAB = endoscopic atraumatic coronary artery; NA = not available; RCT = Randomized Controlled Trial; TVD = triple-vessel disease; LAD = left anterior descending artery; RCA = right coronary artery

### Incidence of MACCE

There was no significant difference in perioperative (≤ 30 days) MACCE between HCR and CABG (OR = 0.90, 95% CI 0.54–1.48, p = 0.67). MACCE were followed up as time went on. 11.3% patients treated with HCR and 8.9% patients with CABG had suffered from MACCE during one-year follow-up (30 days to 1 year) (OR = 1.35, 95% CI 0.73–2.49, p = 0.34). A total of 13.7% patients treated with HCR and 8.7% patients who had undergone CABG have suffered from at least one kind of the MACCE during mid-term follow-up (1 year to 5 years) (OR = 1.25, 95% CI 0.53–2.97, p = 0.61). MACCE occurred in 28.6% patients after HCR and 30.2% patients after CABG during the long-term follow-up (5 years and above) (OR = 0.93, 95% CI 0.61–1.41, p = 0.72). The outcomes of MACCE at short-term, mid-term and long-term follow-up were not statistically dissimilar. Detailed data are shown in Table 2, Table 3.

**Table 2:** Summary of In-hospital Outcomes Comparing HCR and CABG

Subgroup	No. of studies	OR	95%CI	*p*	I^2^ (%)	Effect-model
MACCE	6	0.90	0.54–1.48	0.67	0	Fixed
ICU LOS	9	− 13.34	− 20.27 to (− 6.41)	< 0.001	95	Random
Ventilation time	5	− 8.69	− 17.74 to 0.36	0.06	97	Random
Major bleeding	2	0.36	0.11–1.22	0.10	58	Fixed
Return to work	2	− 68.26	− 77.99 to (− 58.53)	< 0.001	0	Fixed
Atrial fibrillation	8	0.58	0.36–0.93	0.02	56	Random
Infection	6	0.24	0.09–0.64	0.004	1	Fixed
cTnI	3	− 0.39	− 1.06 to 0.27	0.25	83	Random
25% increase in creatinine	2	0.88	0.55–1.39	0.57	0	Fixed
Cerebrovascular accidents	2	0.87	0.21–3.65	0.85	42	Fixed
Mechanical ventilation > 24 h	5	0.49	0.32–0.76	0.001	31	Fixed
Any transfusion of packed red blood cells	10	0.38	0.28–0.51	< 0.001	58	Random
Neurologic event	2	1.24	0.15–10.53	0.84	0	Fixed
Renal failure	7	0.72	0.40–1.31	0.29	51	Random
Operation time	3	− 27.86	− 109.08 to 52.37	0.50	99	Random
Extubation in operating room	2	0.13	0.04–0.41	< 0.001	0	Fixed
Death	8	1.65	0.90–3.02	0.11	0	Fixed
Myocardial infarction	12	0.77	0.45–1.30	0.32	0	Fixed
Pleural effusion	2	0.44	0.20–0.97	0.04	0	Fixed
Repeat revascularization	6	3.50	2.07–5.94	< 0.001	0	Fixed
Hemodialysis	3	0.3	0.05–1.75	0.18	0	Fixed
Platelet transfusion	2	0.41	0.18–0.91	0.03	0	Fixed
Major complications	3	0.40	0.16–1.03	0.06	61	Random
Reopening for bleeding	8	1.11	0.80–1.53	0.53	0	Fixed
Stroke	7	0.92	0.44–1.94	0.84	0	Fixed
Hospital length of stay	11	− 1.62	− 2.38 to (− 0.85)	< 0.001	87	Random

HCR, hybrid coronary revascularization; CABG, coronary artery bypass grafting; OR, odds ratio; CI, confidence interval; MACCE,major adverse cardiac or cerebrovascular events; ICU LOS, intensive care unit length of stay

The effect measure is standard mean difference (SMD): cTnI. The effect measure is weighted mean difference (WMD): ICU LOS; Ventilation time; Return to work; Operation time; Hospital length of stay

**Table 3:** Summary of Follow up Outcomes Comparing HCR and CABG

Subgroup	No. of studies	OR	95%CI	*p*	I^2^ (%)	Effect-model
One-year						
MACCE	6	1.35	0.73–2.49	0.34	0	Fixed
Death	5	1.32	0.47–3.70	0.59	0	Fixed
Myocardial infarction	6	1.31	0.64–2.70	0.46	0	Fixed
Repeat revascularization	6	3.28	1.62–6.64	< 0.001	0	Fixed
Stroke	2	3.73	0.60–23.02	0.16	0	Fixed
Within one to five year						
MACCE	4	1.25	0.53–2.97	0.61	64	Random
Neurologic event	2	0.29	0.07–1.15	0.08	22	Fixed
Death	3	1.90	0.66–5.48	0.24	0	Fixed
Myocardial infarction	3	1.18	0.44–3.14	0.74	33	Fixed
Repeat revascularization	4	2.84	1.64–4.92	< 0.001	0	Fixed
Five-year						
MACCE	3	0.93	0.61–1.41	0.72	25	Fixed
Death	3	0.35	0.18–0.69	0.002	15	Fixed
Myocardial infarction	3	0.83	0.34–2.04	0.68	0	Fixed
Repeat revascularization	4	1.05	0.71–1.53	0.82	7	Fixed
Stroke	2	0.57	0.16–2.01	0.39	0	Fixed

HCR, hybrid coronary revascularization; CABG, coronary artery bypass grafting; OR, odds ratio; CI, confidence interval; MACCE, major adverse cardiac or cerebrovascular events; ICU LOS, intensive care unit length of stay

### Secondary outcomes

Twelve studies referred to the MI. No significant difference was observed to the incidence of MI between HCR group and CABG group during perioperative period (≤ 30 days) (OR = 0.77, 95% CI 0.45–1.30, p = 0.32); short-term (30 days to 1 year) (OR = 1.31, 95% CI 0.64–2.70, p = 0.46), mid-term (1 year to 5 years) (OR = 1.18, 95% CI 0.44–3.14, p = 0.74) and long-term follow-up (5 years and above) (OR = 0.83, 95% CI 0.34–2.04, p = 0.68). Death was reported in eight studies. The mortality in perioperative period (OR = 1.65, 95% CI 0.90–3.02, p = 0.11), short-term (OR = 1.32, 95% CI 0.47–3.70, p = 0.59) and mid-term follow-up (OR = 1.90, 95% CI 0.66–5.48, p = 0.24) between HCR group and CABG group were not significant. Three articles have mentioned the death during long-term follow-up. Patients who had undergone HCR had a lower risk of mortality rate during long-term follow-up compared with those who had undergone CABG (OR = 0.35, 95% CI 0.18–0.69, p = 0.002) (Table 2, Table 3).

Seven articles mentioned repeat revascularization. HCR was associated with a significantly higher risks for repeat revascularization in perioperative period (OR = 3.50, 95% CI 2.07–5.94, p < 0.001), short-term (OR = 3.28, 95% CI 1.62–6.64, p < 0.001)and mid-term follow-up (OR = 2.84, 95% CI 1.64–4.92, p < 0.001). But the prevalence of long-term follow-up repeat revascularization was not significantly different between the two groups (OR = 1.05, 95% CI 0.71–1.53, p = 0.82). Patients treated with HCR did not display a significant reduction in risk of developing a stroke as compared to those who underwent CABG in perioperative period (OR = 0.92, 95% CI 0.44–1.9, p = 0.84), short-term (OR = 3.73, 95% CI 0.60–23.02, p = 0.16) and long-term follow-up (OR = 0.57, 95% CI 0.16–2.01, p = 0.39) (Table 2, Table 3).

Table 2 shows the comparison in in-hospital outcomes. The need for any transfusion of packed red blood cells was significantly lower in the HCR group (OR = 0.38, 95% CI 0.28–0.51, p < 0.001). Moreover, patients in HCR group were associated with a significantly shorter ICU stay (WMD = − 13.34, 95% CI − 20.27 to (− 6.41), p < 0.001) and hospital stay (WMD = − 1.62, 95% CI − 2.38 to (− 0.85), p < 0.001) on average. Results were not significantly different when it comes to development of renal failure (OR = 0.72, 95% CI 0.40–1.31, p = 0.29). The event rate of having AF (OR = 0.58, 95% CI 0.36–0.93, p = 0.02) (Table 2) and infection(OR = 0.24, 95% CI 0.09–0.64, p = 0.004) was higher in CABG group compared with HCR group.

## Discussion

An ideal coronary revascularization should offer minimal invasiveness and maximal durability in order to reduce the risk of surgery and increase the survival rate [7, 14]. Under the circumstances, HCR is an alternative for patients with MVD. In this meta-analysis from 15 observational cohort studies and 3 RCTs, we focused on occurrence of MACCE and its components (MI, stroke, mortality, and repeat revascularization) over time. By reviewing the previous meta-analysis, similar conclusions have been raised. To be more detailed, HCR have similar short-term results and lower blood transfusion rates and infection rates than CABG, which our results confirmed again [15]. In addition to the parameters mentioned, which were provided detailly in previous literatures and recent meta-analysis studies, long-term (5 years and above) results in terms of efficacy of HCR vs CABG for treating MVD were assessed by our team for the first time. The outcomes suggested that HCR did not only merit over CABG in short-run, but also had a trend of better long-term results, particularly in survival aspect.

MACCE is the composite endpoint of MI, stroke, mortality, and repeat revascularization. No significant between-group difference of MACCE during the follow-up period has been discovered. However, HCR tended to be hold a lower MACCE incidence in the long-term follow-up. This is possibly because, patients with CABG is superior for repeated revascularization in the short-term follow up while patients with HCR seems to be associated with lower perioperative MI and stroke rates, though not statistically significant. In the long-term follow-up, there is no difference between the two groups in repeated revascularization as well as death, HCR patients have obvious advantages. This outcome is consistent with a prospective randomized pilot study, HYBRID, which is the only randomized trial worldwide with a long-term follow-up [18]. Moreover, previous meta had supported this view. Hu et al. [17] and Sardar et al. [43] respectively revealed that there was no difference in MACCE incidence between HCR group and CABG group in both short-term and mid-term follow-up.

The possible reasons for the advantage in CABG group in early repeated revascularization were as follows. (1) Stents cannot (but bypass grafting can) prevent disease progression near the injured site [44]. (2) Long term Computer Tomography(CT) follow-up indicated that the incidence of primary artery occlusion in MVD patients after transplantation was 3 times higher than that those without transplantation. Thus, due to insufficient target vessels, the progression of coronary heart disease may be an obstacle in of vascular reconstruction in patients after CABG [45]. (3) Due to the recognized low rate of revascularization and increased invasiveness of CABG, patients who presents early atypical chest symptoms after sternotomy may be diagnosed with incision pain, musculoskeletal or pericardial disease and requires medication. Atypical symptoms after PCI are more likely to bring concern about disease progression or stent restenosis. Thus, the possibility of coronary angiography is increased, which has also been demonstrated. Hage et al. [28] pointed out that patients who have underwent CABG did not undergo any routine angiographic evaluation of the graft, while all HCR patients were required with postoperative angiograms, and any patients found with significant anastomotic abnormalities underwent would be required to undergo intervention. Therefore, the rates of graft failure and revascularization in the CABG group may have been underestimated. But there is no significant difference in long-term follow-up. This may due to increase in the risk of saphenous vein graft(SVG) degeneration after the intervention period over time. The graft occlusion rate is approximately 20% in the first year, 30% in 10 years, and almost 70% in 15 years [46]. Non-LAD vascular PCI does not depend on saphenous vein transplantation. Repeat interventions due to stent failure usually occur in the first few years after surgery. With the development of newer generation DES, restenosis rates have been decreased to < 10% overall and < 5% after one year [47]. In summary, non-LAD vessels underwent CABG will present a progressively higher stenosis rate over time while underwent PCI will increase stent stenosis rate slowly in the long term, hence there will be no difference in late repeat revascularization.

Our data seems to have revealed a more pronounced ascent trend in mortality rate in CABG group compared with HCR group as follow-up duration increased. There was no significant difference in perioperative period; short-term, mid-term follow-up between patients underwent HCR and CABG respectively. However, in the long-term follow-up, patients who had undergone HCR had a drastic advantage over those who had undergone CABG. The speculative mortality trends conclusion raised by another study is in accordance with ours [48]. This may be due to the following reasons. (1) A staged CABG-then-PCI strategy, the current most common approach, enable confirm the quality of LIMA–LAD graft at the time of PCI thus defects can be corrected immediately in time [49]. (2) Aortic clamping is avoided during HCR but it is necessary during on-pump CABG, which may increase the risk of cerebral infarction [50]. (3) We recorded the all-cause death, so deaths may not necessarily have occurred by cardiovascular events. Compared with CABG, HCR is less invasive and less harmful to other body systems, which is especially beneficial for elderly patients. (4) we speculate that it is the difference of severity in MACCE that leads to similarity of occurrence probability and differences in death. Previous studies have shown that after stents intervention, angina is reduced, and stroke is mainly in the early stage [28]. Regrettably, no studies have been done to determine whether there is a difference in MACCE degree between these two. (5) Death is a clear indicator for statistical convenience, while MACCE is a mixed event, and leading to errors in assessments. However, Harskamp et al. [51] concluded in a meta-analysis that there was no difference in mortality rate between the two groups. He illustrated that the main concern with HCR is the quality of the anastomosis, especially the long-term adverse clinical outcome is related to LIMA deficiency. Nevertheless, the failure rate of LIMA after HCR was significantly higher than that of CABG in the long-term follow-up. However, LIMA failure is considered to be more related to repeat revascularization and has no direct link with mortality. Harskamp et al. also have only included six observational studies which definitely present a large risk of bias due to the limited amount of literature. This can result from many more patients have appeared with more severe disease that refuses CABG due to the more invasive nature [43]. Therefore, his conclusion is considered to be controversial and awaits further confirmation.

Besides, there was no difference in stroke and MI in each period between two groups. This conclusion is consistent among all existing RCTs [25, 26]. Sardar et al. [43] indicated that, comparing with traditional coronary artery bypass grafting, the risk of stroke after arterial embolization is lower in theory. Our data also showed a lower but not statistically significant perioperative risk for stroke in the HCR group, and this may be because the avoidance of aortic valve surgery and cardiopulmonary bypass in HCR. The atherosclerotic plaque displacement during aortic surgery has been considered as the major cause of stroke [52]. Also, there is a significant difference in the degree of cardiac surgery between HCR and CABG. The heart is in a natural position in HCR, but needs to be rotated frequently in CABG, which may affect the hemodynamic state. In addition, the stent placement time is less than 30 s, thus coronary artery occlusion during HCR is limited to the one that requires to place a single LIMA-LAD graft. In contrast, CABG requires a 8–12 min of coronary artery occlusion in each of the three or four distal anastomoses, resulting in a total ischemic time of estimately 25–40 min [17].

In a large number of randomized trials, the combination of clopidogrel and aspirin has been proved to drastically reduce the incidence of stent thrombosis in PCI patients [53]. Together with antiplatelet therapy, HCR avoids sternotomy and is less invasive, compared with CABG. These advantageous characteristics of HCR are widely believed also by other peer researchers to be capable to result in lower blood transfusion rate, shorter ventilation time and shorter ICU as well as other kinds of, hospital stay in HCR group. Patients are able to have a higher quality of life and greater satisfaction [28, 29].

We found that the rate of AF was relatively low in the perioperative period of HCR. A series of reports investigated that the incidence of AF after minimally invasive CABG (MIDCAB) is between 4 and 23% [29]. These differences may result from subtle though unidentified differences in surgical or anesthetic techniques. As there will be fine precautions designed in advance to control bleeding during MIDCAB, the pericardial window into LAD should be as small as possible, leading to a low incidence of postoperative AF.

In general, HCR has better perioperative results with a good trend of long-term survival rate, but its repeat revascularization rate is higher in the short- and medium-term period. Although repeat revascularization is often evaluated, it is far less important for patients than other endpoints, such as death, MI. It is a complicated to define whether repeating revascularization for patients with stable condition is essential or not. Among all the influential factors, the most vital ones are instructions and preferences, while other endpoints are more qualitative rather than quantitative assessed [53]. Therefore, even if HCR has a higher revascularization rate in the short- and medium- term perspective, its feasibility can still be proposed in treatments for MVD patients.

Though in theory, HCR combines the advantages of both procedures, it is still suggested that HCR actually combines the unfavorable disadvantages. For example, performing two types of invasive surgery in a short period of time requires a high degree of cooperation between the two surgical teams, not mentioning the higher cost. Therefore, HCR needs to be generalized with greater advantages in follow-up.

Our study also has some limitations. Firstly, there are only three RCTs compareing the safety of HCR and CABG while more observational studies have been included. Therefore, chaos between the trial population and selection bias may affect the results. Secondly, the definition of MACCE varies from study to study. Most studies define MACCE as a composite endpoint of MI, stroke, mortality, and repeat revascularization. Others replace repeat revascularization with target vessel revascularization (TVR), or delete stroke. After all, there are few studies with different definitions, hence the general speculation is not significant.

## Conclusion

In summary, HCR is relatively less invasive with better perioperative results, and hold a higher trend in long-term survival rate for MVD patients. HCR has the same therapeutic effect but lower long-term mortality incidence compared with CABG. Therefore, we believe that HCR is a promising alternative methods for MVD patients though more long-term RCTs with large-scale are needed in the future.

## Supplementary Information

**Additional file 1: Table S1**. Characteristics of all the studies included in the meta-analysis.

**Additional file 2: Table S2**. Quality assessment of studies included.

## Data Availability

Data supporting findings reported in this study are available in the supplementary materials.

## Abbreviations

PCI: Percutaneous coronary intervention
CABG: Coronary artery bypass grafting
MVD: Multivessel coronary artery disease
HCR: Hybrid coronary revascularization
MACCE: Major adverse cardiovascular and cerebral events
RCT: Randomized controlled trial
CVD: Cardiovascular diseases
CAD: Coronary artery disease
LMCA: Left main coronary artery
LIMA: Left internal mammary artery
LAD: Left anterior descending
ICU: Intensive care unit
AF: Atrial fibrillation
DES: Drug-eluting stent
MI: Myocardial infarction
MIDCAB: Minimally invasive CABG
OR: Odds ratio
CI: Confidence interval
SMD: Standardized mean difference
WMD: Weighted mean difference
CT: Computer Tomography
