# Supplementary material for: Comparison of hybrid coronary revascularization versus coronary artery bypass grafting in patients with multivessel coronary artery disease: a meta-analysis
Source: J Cardiothorac Surg. 2022 Jun 7;17:147. doi: 10.1186/s13019-022-01903-w (PMC9175312; doi:10.1186/s13019-022-01903-w)
Supplement: Supplementary file 1 — Additional file 1: Table S1. Characteristics of all the studies included in the meta-analysis. [file 13019_2022_1903_MOESM1_ESM.docx]

**Supplementary Table 1. Characteristics of all the studies included in the meta-analysis.**

| Author | Year | Age | | Diabetes(%) | | LVEF(%) | | Study period | Antiplatelet strategy |
| --- | --- | --- | --- | --- | --- | --- | --- | --- | --- |
|  |  | HCR | CABG | HCR | CABG | HCR | CABG |  |  |
| Hage | 2019 | 61.7 | 68.8 | 22 | 28 | NA | NA | 2004.3-2015.11 | NA |
| Patel | 2018 | 65 | 65 | 47.3 | 47.4 | 52.3 | 50.5 | 2009.1-2016.12 | NA |
| Qiu | 2019 | 64.6 | 62.6 | 44.2 | 26.6 | NA | NA | 2009.1-2016.12 | aspirin (100 mg/ day) and clopidogrel (75 mg/day) |
| Wu | 2017 | 61.1 | 63.1 | 35.6 | 35 | 60.0 | 59.3 | NA | aspirin (100 mg/ day) and clopidogrel (75 mg/day) |
| Di Bacco | 2019 | 64 | 74 | 26.2 | 25.6 | NA | NA | 2000-2015 | NA |
| Hannan | 2020 | 65.79 | 65.28 | 37.09 | 41.72 | 53.36 | 53.78 | 2010.1-2016.11 | NA |
| Shen | 2013 | 62 | 62.4 | 26.2 | 18.4 | 62.7 | 62.6 | 2007.6-2010.12 | aspirin (300 mg/ day) and clopidogrel (75 mg/day) |
| Modrau | 2020 | 68.3 | 68.3 | 16 | 12 | NA | NA | 2010.10-2012.2 | NA |
| Basman | 2020 | 67.5 | 65.8 | 53 | 55.6 | 53.3 | 48.8 | 2009.1-2016.12 | NA |
| Zhao | 2009 | 63 | 63 | 39 | 39 | 50 | 54 | 2005.4-2007.6 | aspirin (325 mg/ day) |
| Delhaye | 2010 | 62 | 60 | 44.5 | 38.9 | 60 | 60 | 2006.10-2008.1 | aspirin (160 mg/ day) and clopidogrel (75 mg/day) |
| Harskamp | 2015 | 64.6 | 64.8 | 36.9 | 39 | 54.7 | 54.5 | 2003.10-2013.9 | NA |
| Kon | 2008 | 61 | 65 | 27 | 40 | 47 | 45 | 2005.1-2016.12 | aspirin (325 mg/ day) and clopidogrel (75 mg/day) |
| De Cannière | 2001 | 62 | 63 | 20 | 25 | 56 | 55 | 1997.1-1997.12 | NA |
| Farid | 2018 | 64 | 61 | 10 | 13 | NA | NA | 2001.1-2015.12 | aspirin (300 mg/ day) and clopidogrel |
| Gąsior | 2014 | 63.1 | 63.9 | 25.5 | 30.4 | 49.8 | 50.7 | NA | aspirin and clopidogrel |
| Ganyukov | 2020 | 62 | 61.3 | 17.3 | 22 | 56.2 | 54 | NA | aspirin (75 mg/ day) and clopidogrel (75 mg/day) |
| Esteves | 2020 | 61.1 | 61.1 | 37.5 | 50 | 59.8 | 61.3 | NA | aspirin (100 mg/ day) and clopidogrel (75 mg/day) |

CABG, coronary artery bypass grafting; HCR, hybrid coronary revascularization; LVEF, left ventricle ejection fraction; NA, not available.
