# Supplementary material for: Comparison of hybrid coronary revascularization versus coronary artery bypass grafting in patients with multivessel coronary artery disease: a meta-analysis
Source: J Cardiothorac Surg. 2022 Jun 7;17:147. doi: 10.1186/s13019-022-01903-w (PMC9175312; doi:10.1186/s13019-022-01903-w)
Supplement: Supplementary file 2 — Additional file 2: Table S2. Quality assessment of studies included. [file 13019_2022_1903_MOESM2_ESM.docx]

**Supplementary Table 2. Quality assessment of studies included.**

| Author, year,  Study (RCT) | Sequence  Generation | Allocation  Concealment | | Blinding | Incomplete  outcome data | | Selective  outcome reporting | | Free of  other bias |  |  |  |  |  |
| --- | --- | --- | --- | --- | --- | --- | --- | --- | --- | --- | --- | --- | --- | --- |
| Gąsior, 2014 | low risk | low risk | | low risk | low risk | | low risk | | unclear risk |  |  |  |  |  |
| Ganyukov, 2020 | low risk | low risk | | high risk | low risk | | low risk | | unclear risk |  |  |  |  |  |
| Esteves, 2020 | low risk | low risk | | high risk | low risk | | low risk | | unclaer risk |  |  |  |  |  |
| Author, year,  Study (Observational) | **Selection (Out of 4)** | | | | | | | | | **Comparability**  **(Out of 2)** | **Outcomes (Out of 3)** | | | **Total**  **(Out of 9)** |
|  | Representativeness of exposed cohort | | Selection of nonexposed cohort | | | Ascertainment  of exposure | | Outcome not present at the start of the study | |  | Assessment of outcomes | Length of follow-up | Adequacy of follow-up |  |
| Hage, 2019 | 1 | | 1 | | | 1 | | 1 | | 2 | 1 | 1 | 1 | 9 |
| Patel, 2018 | 1 | | 1 | | | 1 | | 1 | | 2 | 1 | 1 | 1 | 9 |
| Qiu, 2019 | 1 | | 0 | | | 1 | | 1 | | 2 | 1 | 1 | 1 | 8 |
| Wu, 2017 | 1 | | 1 | | | 1 | | 1 | | 2 | 0 | 1 | 0 | 7 |
| Di Bacco, 2019 | 1 | | 0 | | | 1 | | 1 | | 1 | 1 | 1 | 1 | 7 |
| Hannan, 2020 | 1 | | 1 | | | 0 | | 1 | | 2 | 1 | 1 | 1 | 8 |
| Shen, 2013 | 1 | | 1 | | | 1 | | 1 | | 2 | 1 | 1 | 1 | 9 |
| Modrau, 2020 | 0 | | 1 | | | 1 | | 1 | | 2 | 1 | 1 | 1 | 8 |
| Basman, 2020 | 1 | | 1 | | | 1 | | 1 | | 1 | 1 | 1 | 1 | 8 |
| Zhao, 2009 | 1 | | 1 | | | 1 | | 1 | | 2 | 1 | 1 | 1 | 9 |
| Delhaye, 2010 | 1 | | 0 | | | 1 | | 1 | | 2 | 1 | 0 | 1 | 7 |
| Harskamp, 2015 | 1 | | 1 | | | 1 | | 1 | | 2 | 1 | 1 | 1 | 9 |
| Kon, 2018 | 1 | | 0 | | | 1 | | 0 | | 2 | 1 | 1 | 1 | 8 |
| DeCannière, 2001 | 0 | | 1 | | | 1 | | 1 | | 1 | 1 | 1 | 1 | 7 |
| Giambruno, 2018 | 1 | | 1 | | | 1 | | 1 | | 2 | 1 | 1 | 1 | 9 |
| Farid, 2018 | 1 | | 1 | | | 1 | | 1 | | 1 | 1 | 1 | 1 | 8 |

The RCTs and observational studies were assessed by the Cochrane Collaboration’s tool and Newcastle-Ottawa Quality Assessment Scale, respectively.

Risk of bias was assessed as “low risk”, “high risk” or “unclear risk”.
